# Supplementary material for: Improvement of a synthetic live bacterial therapeutic for phenylketonuria with biosensor-enabled enzyme engineering
Source: Nat Commun. 2021 Oct 28;12:6215. doi: 10.1038/s41467-021-26524-0 (PMC8553829; doi:10.1038/s41467-021-26524-0)
Supplement: Supplementary file 1 — Supplementary Information [file 41467_2021_26524_MOESM1_ESM.pdf]

Rationale behind PAL enzyme engineering

E. coli Nissle 1917 (EcN) strains engineered for the therapeutic treatment of phenylketonuria (PKU) express the gene encoding phenylalanine ammonia lyase (PAL) to convert exogenous phenylalanine (Phe) to *trans*-cinnamate (TCA). The work described here encompasses the efforts undertaken to improve PAL activity in the context of whole cells. Preliminary work aimed to determine if strain activity was rate-limited by PAL expression, as this would offer a simple route for strain optimization. However, data indicated that increasing PAL expression in EcN strains did not result in increased PAL activity, while, conversely, activities were increased in their associated lysates (Supplementary Fig. 1a). SDS PAGE gels further supported that PAL abundance in whole cells did not correlate with whole cell strain activity (Supplementary Fig. 1b). Because the activity of PAL in whole cell context may be complex and rate-limited by a variety of physiological and/or environmental factors, we sought a top-down directed evolution approach for strain improvement that did not require prerequisite knowledge of whole cell rate-limitation.

a

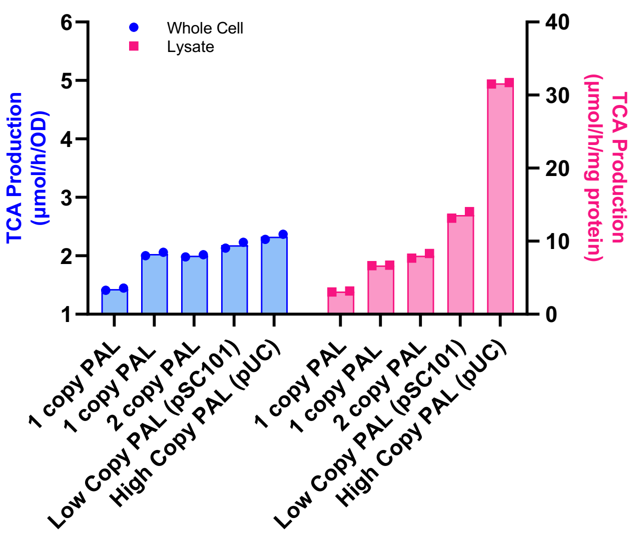

b

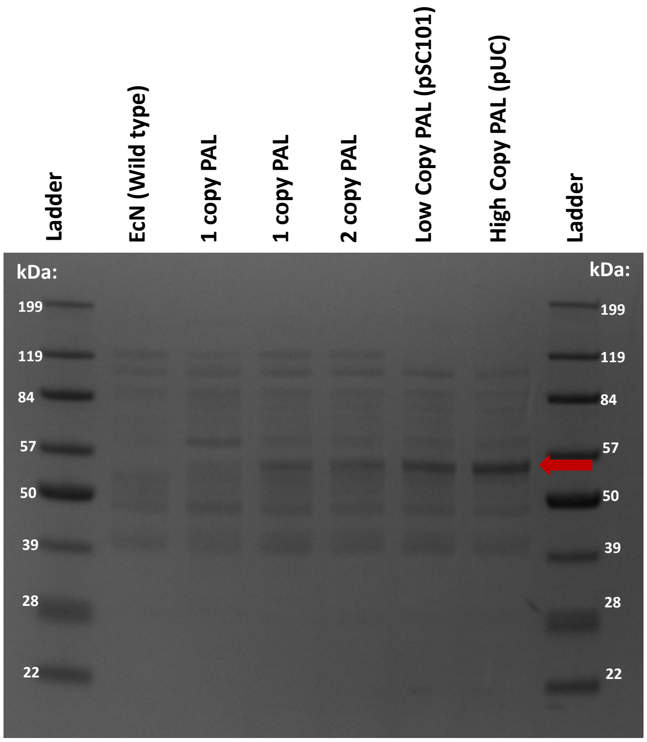

Supplementary Fig. 1. EcN strains were engineered to express PAL. The engineered strain series include strains (2) with a single chromosomal insertion of *stlA* (encoding PAL) in separate locations (1 copy PAL), a strain containing both *stlA* gene copies in the same background (2 copy PAL), a strain with a low copy plasmid expressing *stlA* (Low Copy PAL (pSC101)), and a strain with a high copy plasmid expressing *stlA* (High Copy PAL (pUC)). In each of these strains, the same *stlA* coding sequence, ribosome binding site, and anhydrotetracycline-inducible promoter was used so that the only difference between strains was the locations of chromosomal insertion and/or *stlA* copy number. **a,** The blue bars represent whole cell

PAL activity from strains (left *y*-axis; activity normalized to cell number) while the pink bars represent the corresponding PAL activity from lysates generated from the same cell batches (right *y*-axis; activity normalized to total soluble protein content from clarified lysate as determined by BCA assay). The assay was performed in biological duplicate. **b,** SDS PAGE gels were run using whole cells of the strain series described above. Briefly, strains were grown and induced for PAL activity as described in Methods. Whole cells were resuspended to an OD_{600} of 0.5, boiled in lithium dodecyl sulphate (LDS) sample buffer for 15 minutes, and loaded onto a 4-12% Bis-Tris SDS PAGE stacking gel. Compared to EcN, a novel band was apparent running slightly under the 57 kDa protein standard, consistent with the approximate size of PAL (red arrow). This band, presumed to be PAL, was shown to increase in abundance with increasing copy number. This experiment was repeated twice and yielded similar results each time.

Pop ‘n’ sort methodology

Pop ‘n’ sort offers the diffusion mitigation of water-in-oil encapsulation with the ease, throughput, and commercial availability of fluorescence activated cell sorters (FACS). The benefit of using encapsulation during simultaneous growth, production, and sensing is highlighted below in a proof-of-concept sorting experiment performed on an ~300-member library of PAL variants (see description in “Engineering Rounds” below, Round 2). Using a Welch’s ANOVA test with Dunnett’s T3 multiple comparisons test to assess significance, enrichment was successful when using the pop ‘n’ sort strategy (mock-mock vs. top 1%-top 1%, $p = 0.0007$) but not when sorting from liquid cultures (mock-mock vs. top 1%-top 1%, $p = 0.1408$). Additionally, the top 1%-top 1% pool is significantly improved above wild-type when using pop ‘n’ sort ($p = 0.0073$), but not when sorting from liquid culture ($p > 0.9999$).

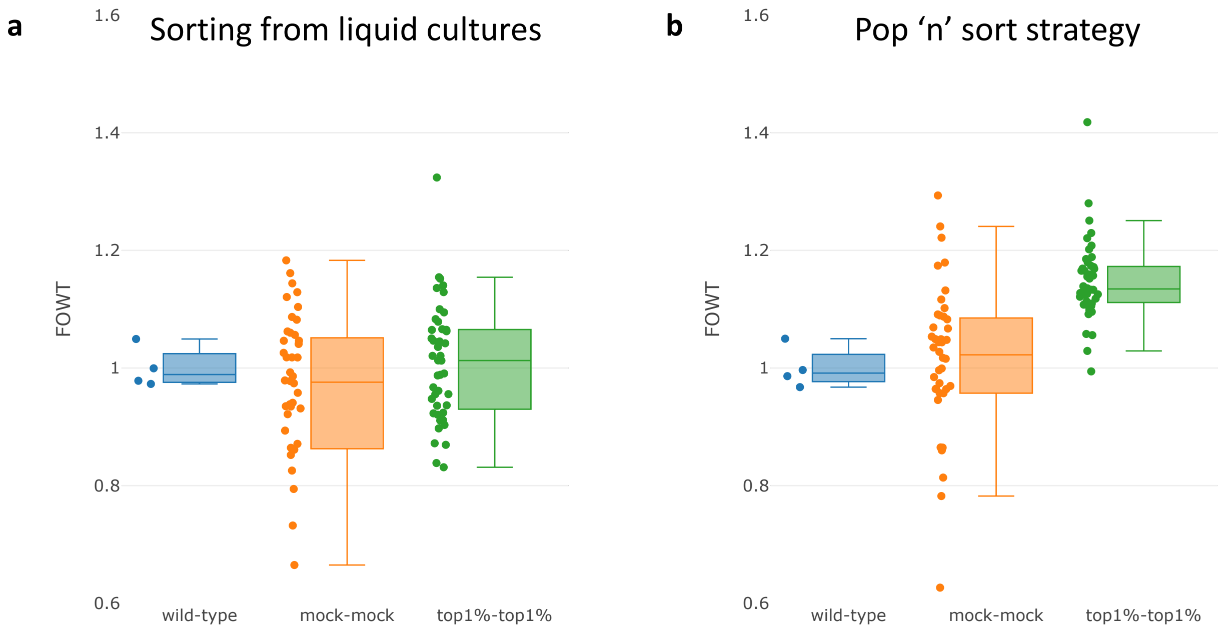

Supplementary Fig. 2. Liquid screening vs. pop ‘n’ sort. A small-scale ~300-member combinatorial library of *stlA* variants was assayed using either a liquid culture approach (**a**) or pop ‘n’ sort (**b**). Sorting from liquid cultures followed the same protocol as pop ‘n’ sort (Methods, Fig. 2), except that the cells

were never encapsulated, and instead were simply incubated with shaking following the washing and resuspension step. Following enrichment, colonies were assayed using the plate-based activated biomass protocol described in the Methods. For each of the two sorting strategies, n=4 wild-type PAL biological replicates (blue), n=44 independent colonies from a mock-mock sort (orange), and n=44 independent colonies from a top 1%-top 1% sort (green) were assayed in parallel (one 96-well plate for the liquid sort, and one 96-well plate for pop 'n' sorted). Boxplots with accompanying clonal data points, showing FOWT of TCA produced in 4 h. Mock-mock: two sequential mock sorts (sorted based on cell size and doublet exclusion without GFP-based gating). Top 1%-top 1%: two sequential top 1% sorts (sorted based on cell size and doublet exclusion as well as the top 1% brightest events). Boxes extend from the first to third quartile, with a center line indicating the median. Upper and lower whiskers extend to the maximum and minimum point, respectively, within 1.5 times the interquartile range of the bounds of the box. Data points outside of the whiskers are considered outliers. A single low activity outlier was excluded from the mock-mock population of panel b for improved data visualization, and can be found in the accompanying Source Data.

Engineering rounds

The focus of this Article is on a single round of PAL engineering, the enrichment of a >1 million-member library, and the identification of an improved PAL variant that was further developed for use in a clinical strain. As alluded to in the discussion of Fig. 1b as well as the >1 million-member library Methods and discussion, early efforts were made to engineer the enzyme ahead of the successful screening of a large library. Supplementary Fig. 3 below shows the activity relative to wild-type (fold over wild-type, FOWT) of the 411 unique variants that were identified throughout the course of the Synlogic-Zymergen collaboration, separated into multiple rounds of engineering.

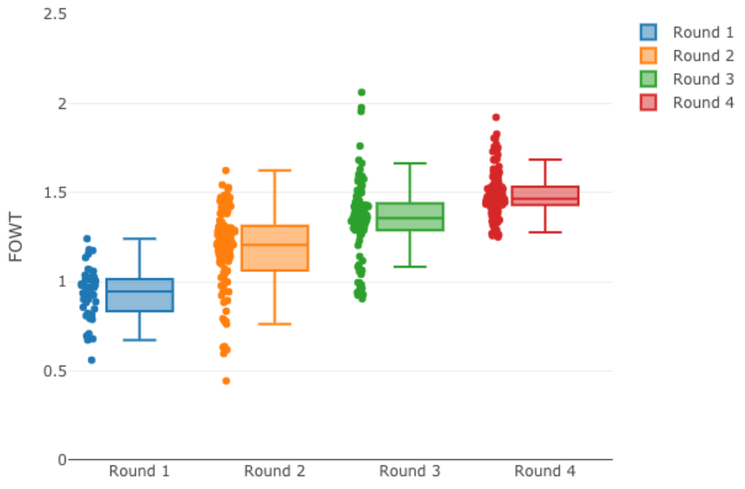

Supplementary Fig. 3. Unique variants identified throughout project. Fold over wild-type (FOWT) 4 h whole cell activity of 411 unique PAL variants identified throughout the Synlogic-Zymergen collaboration (n=48 independent strains in Round 1, n=107 independent strains in Round 2, n=105 independent strains in Round 3, n=151 independent strains in Round 4). Boxes extend from the first to third quartile, with a center line indicating the median. Upper and lower whiskers extend to the maximum and minimum point, respectively, within 1.5 times the interquartile range of the bounds of the box. Data points outside of the whiskers are considered outliers. Each data point represents the activity level of a unique sequenced variant. Where applicable, replicates of the same variant are averaged together, such that a single point appears per variant. FOWT was calculated by normalizing by a wild-type control or controls in the same batch/plate. For much of the data in this plot, replicates were not performed, as we prioritized promising hits over thoroughly characterizing less improved variants.

Round 1 of Supplementary Fig. 3 took place in parallel to the development of a sensor application suitable for large library screening, and largely consisted of clonal assaying of single permutations. These efforts identified a set of neutral and beneficial mutations from early structural, PSSM, and coevolution designs. In Round 2, we combined these neutral and beneficial mutations into the ~300-member library described in Supplementary Fig. 2, which was also the origin of the improved templates for the library discussed in the main text, as well as building out some rational full-length designs (the origin of the strains in Fig. 1b), and strategically layering promising mutations. As shown in Supplementary Fig. 2, by this time we had demonstrated successful library enrichment and were ready to expand our search.

Round 3 consisted of the building and screening of the >1 million-member library described throughout this work, which resulted in top hits with approximately 2-fold improvement over wild-type in whole cell assays. In a final round of engineering (Round 4), we layered additional mutations onto the top 1%-top 1% sorted population described in this work (complexity ≥10,000 variants) in two separate build strategies. On average 2 random mutations per variant were layered onto this pool to generate an ~5 million-member error-prone library. In a separate library, 112 new designed mutations (same approaches as described in the text) as well as 6 optional reversions of the most commonly found mutations (introduced using the inverse PCR approach described in the Methods, but with wild-type sequence) were introduced in a ~14 million-member combinatorial library. Despite identifying many unique improved sequences in this combined ~19 million complexity effort (Supplementary Fig. 2, Round 4), we did not identify variants that surpassed the activity of the top variants identified in Round 3.

Characterization of top candidates in physiological context

Throughout the collaboration, top hits were assayed using a more thorough OD_{600} normalization protocol (described in “Activated biomass assay at various pH or after exposure to low pH” in the Methods, data assayed at neutral pH in Supplementary Table 1), and transferred to Synlogic as appropriate for testing in their *in vitro* simulation (IVS) stomach model (data in Table S1). In addition to those strains, wild-type StlA (EP2315) and variant H133M_I167K (EP2401, an early identified hit) were included as controls. For all the experiments in this section except the initial plate-based measurements in Supplementary Table 1, the variants on low-copy P_{tet}-PAL plasmids were transformed into a fresh wild-type EcN background without sensor. Speculations for how individual mutations in the top variants might be playing a role appear in Supplementary Table 2.

Supplementary Table 1. Variants for more thorough testing.

Summary of the *in vitro* and IVS activity of the 6 StlA variants selected for thorough analysis. Plate-based or tube-based activity refers to activated biomass assays performed at Zymergen, and *in vitro* assay and IVS activity refers to assays performed at Synlogic. Activity is reported as FOWT, in which wild-type refers to EP2315 (EcN with wild-type StlA in the same low-copy P_{tet}-PAL context) activity from the same assay. EP2315 and EP2401 (H133M_I167K, an early hit) are included in this table since these strains served as controls for the analysis of this set of variants. Average +/- s.d. (where available).

Zymergen Strain ID	StlA Mutations	Zymergen production data		Synlogic production data	
		4 h FOWT plate-based assay	4 h FOWT tube-based assay	2 h FOWT *in vitro assay*	2 h FOWT IVS assay*
EP2315	wild-type StlA	1[#]	1[#]	1[#]	1[#]
EP2401	H133M_I167K	-	1.2 +/- 0.05	1.24 +/- 0.08	1.92 +/- 0.17
EP2516	S92G_H133F_A433S_V470A	2.5	2.00 +/- 0.12	1.3	1.29 +/- 0.08
EP2525	S92G_H133M_I167K_L432I_V470A	1.91	1.96 +/- 0.14	2.74	2.29 +/- 0.31
EP2495	A93C_H133F_T322W_Y437N	1.98	1.98 +/- 0.18	1.2	1.05 +/- 0.10
EP2502	I28R_S92G_H133F_V470A	1.49	1.42 +/- 0.08	2.26	2.79 +/- 0.01
EP2528	S92G_H133F_R185E	1.5	1.63 +/- 0.13	2.25	2.24 +/- 0.15
EP2526	S92G_F109A_H133M_T503E	1.31	1.34 +/- 0.12	2.04 +/- 0.08	2.17 +/- 0.17

*Strains grown as described in Methods with the following exceptions: Rather than tangential flow filtration (TFF), cells that had reached stationary phase were centrifuged to collect activated biomass, resuspended in PBS containing 15% glycerol, and stored at -80°C until the day of testing.

[#]This table represents the compilation of FOWT data gathered over the course of months, rather than a single experiment. For the wild-type TCA production reference of each variant FOWT, please refer to the accompanying Source Data.

Supplementary Table 2. Speculations about impact of mutations on activity.

Native residue	Position	Substitution	Hypothesized role / enzyme property	Comments
I	28	R	Charge	I28R might provide an additional charge.
S	92	G	Activity	Located at the C-terminal part of the helix; S92G may serve to increase flexibility by interfering with hydrogen bonding of the carbonyl oxygen. This position is also located near the active site and could influence the binding and release of substrate.
A	93	C	Packing	Might increase packing of enzyme through dipole-quadropole interaction between sulfur of A93C and aromatic ring of F90.
F	109	A	Packing	Change to smaller alanine residue at 109 decreases volume of helix, which could increase helix packing.
H	133	F	Stability, packing	H133F (or H133M) increases packing and helix stability by removing histidine's potential perturbation. H133 could potentially distort a helix by interfering with the carbonyl oxygen from A129.
H	133	M		
I	167	K	Stability	I167K removes an exposed hydrophobic residue, which should improve the stability and biophysical behavior of the enzyme.
R	185	E		No current hypothesis; R185E mutation on its own has a negative impact on enzyme activity.
T	322	W	Stability	T322 is located in the helix; the change to W might increase stability of the enzyme by packing with charged amino acids in its spatial vicinity. It is unclear why W is the preferred substitution.
L	432	I	Activity	L432I changes the phi/psi of loop region by introduction of a beta-branched amino acid. The loop region is near the active site and this change could affect substrate binding and release.
A	433	S	Solvation	A433S increases solvation of the enzyme due to exposed hydroxyl group (small perturbation in the change from A to S).
Y	437	N	Activity	437 is located in the active site: Y437N would increase flexibility and should have an effect on the K_M/k_{cat} of the enzyme.
V	470	A	Stability	Alanine substitution at V470 increases helicity and stability of enzyme.
T	503	E	Solvation	T503E increases solvation energy by introduction of carboxylic acid.

Activity at different assay pH – additional data for Fig. 4b

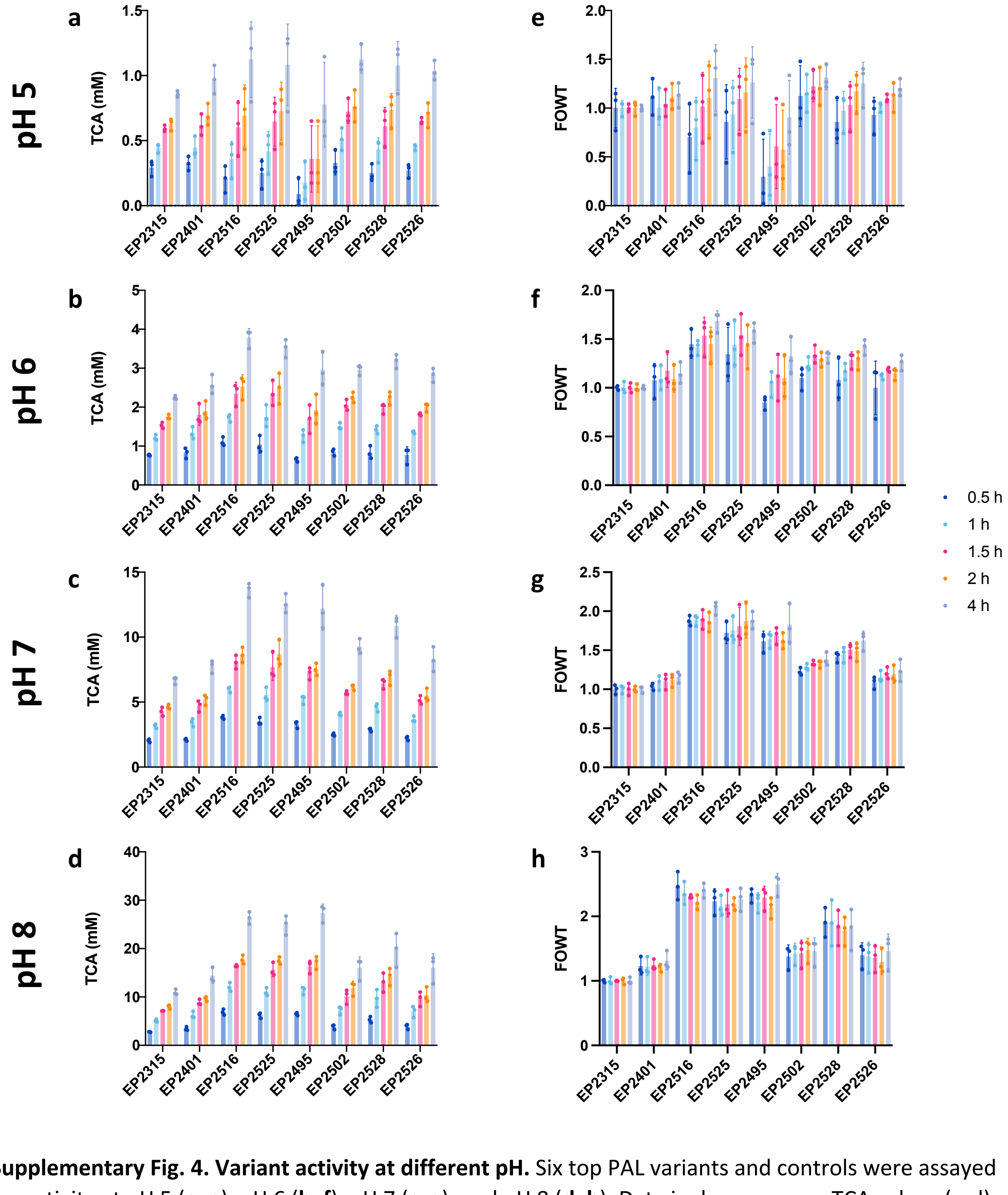

Supplementary Fig. 4. Variant activity at different pH. Six top PAL variants and controls were assayed for activity at pH 5 (**a**, **e**), pH 6 (**b**, **f**), pH 7 (**c**, **g**), and pH 8 (**d**, **h**). Data is shown as raw TCA values (a-d), as well as normalized to wild-type StlA EP2315 for FOWT comparisons (e-h), after 0.5 h, 1 h, 1.5h, 2 h, and 4 h incubation. All bars are shown as average of 3 biological replicates with s.d. error bars and individual data points shown. The 4 h data of panels e-h are identical to that shown in Fig. 4a of the main text.

Recovery of activity after exposure to reduced pH – additional data for Fig. 4c

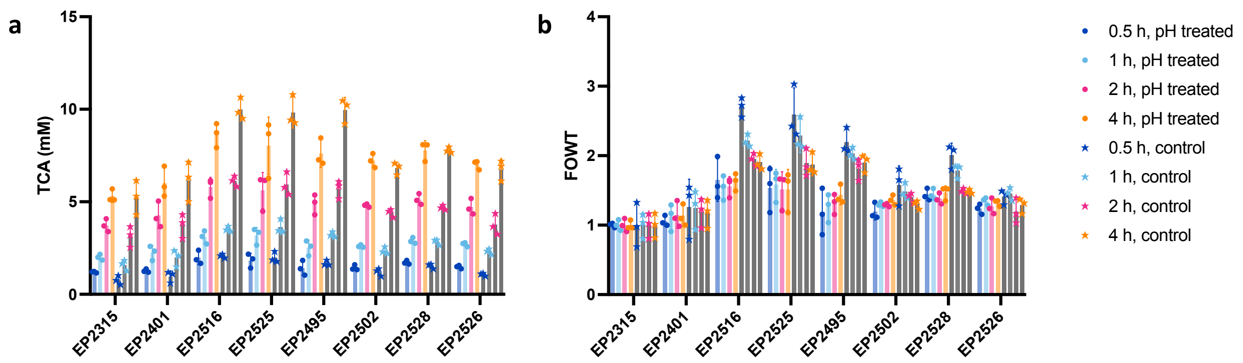

Supplementary Fig. 5. Activity after recovery from acidic environment. a, Raw TCA values for data shown in Fig. 4c and expanded to show additional timepoints (0.5 h, 1 h, 2 h, and 4 h) of activity assay incubation at neutral pH after 1 h incubation at pH 5 or no pre-treatment (controls, gray, account for wash steps, described in the Methods). **b,** Data from panel a represented as fold improvement over wild-type StlA EP2315 (FOWT), and expanded to show additional timepoints compared to panel Fig. 4c. Average of 3 biological replicates with s.d. error-bars and individual data points shown.

Gating strategy example

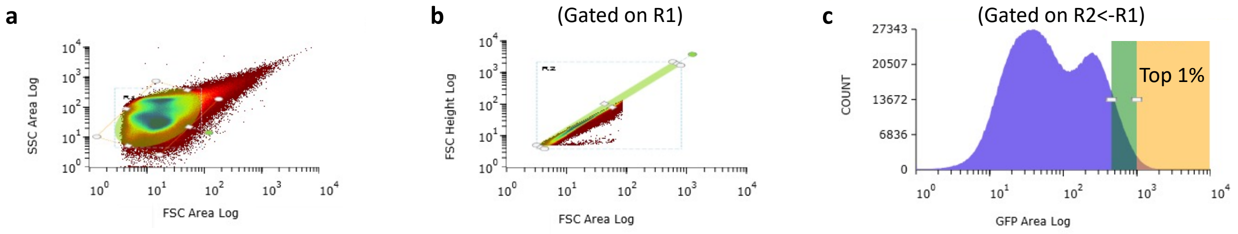

Supplementary Fig. 6. Gating strategy example. A hierarchical gating strategy was applied for sorting. First, an elliptical SSC-Area vs. FSC-Area gate was applied to select cells of a similar size and omit debris (a), followed by gating on FSC-Height vs. FSC-Area to exclude doublets (b, events already gated based on cell size), and finally based on GFP signal (c, events already gated based on cell size and doublet exclusion). In the above example, the orange gate in c corresponds to the top ~1%; the green gate gathers the 1-10% brightest cells, which was not described in this paper. For mock sorts described in the main text, no selection based on GFP was applied.

Kinetic parameter determination of top candidates

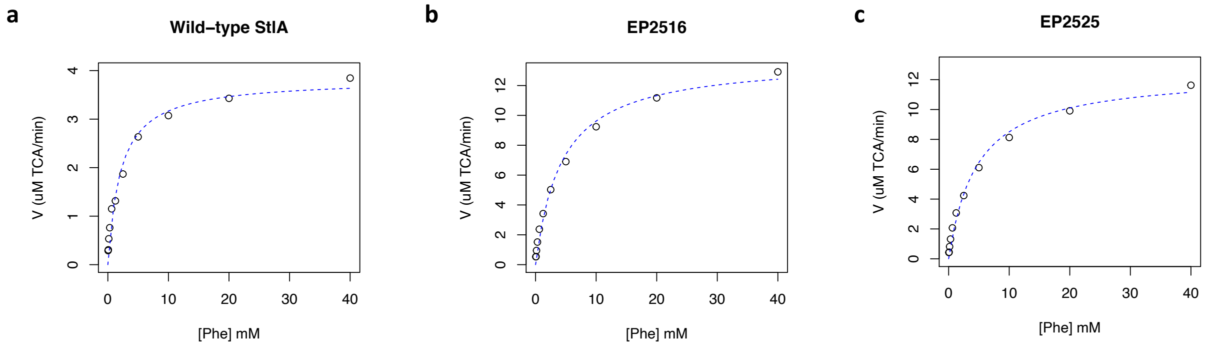

Supplementary Fig. 7. Michaelis-Menten models from lysate kinetic data, example fit. Michaelis-Menten graphs with rate *V* (µM TCA/min) as a function of Phe concentration [Phe] (mM) for wild-type StlA (a) and the two top variants EP2516 (b) and EP2525 (c). The data points on each graph are rate (*V* in µM TCA/min) calculated from the first hour of activity for each Phe concentration tested, where activity remained linear. These plots show an example fit from a single biological replicate out of the three used in generating the values of Table 1.

Viability data for Fig. 5

Supplementary Table 3. Viabilities of EcN strains as determined by SYTOX Green staining and microscopy following resuspension from lyophilization

Strain/Batch	Viability (%)
IVS Assay (Fig. 5b)	
SYN094 (Wild type EcN control)	97.1
SYNB1618	86.6
SYNB1934	97.0
NHP Studies (Fig. 5c and d)	
SYNB1618	86.9
SYNB1934	90.3
