# Supplementary material for: Improvement of a synthetic live bacterial therapeutic for phenylketonuria with biosensor-enabled enzyme engineering
Source: Nat Commun. 2021 Oct 28;12:6215. doi: 10.1038/s41467-021-26524-0 (PMC8553829; doi:10.1038/s41467-021-26524-0)
Supplement: Supplementary file 2 — Reporting Summary [file 41467_2021_26524_MOESM2_ESM.pdf]

nature portfolio

Corresponding author(s): Vincent Isabella

Last updated by author(s): Sep 30, 2021

Reporting Summary

Nature Portfolio wishes to improve the reproducibility of the work that we publish. This form provides structure for consistency and transparency in reporting. For further information on Nature Portfolio policies, see our Editorial Policies and the Editorial Policy Checklist.

Statistics

For all statistical analyses, confirm that the following items are present in the figure legend, table legend, main text, or Methods section.

n/a	Confirmed	
☐	☒	The exact sample size (*n*) for each experimental group/condition, given as a discrete number and unit of measurement
☐	☒	A statement on whether measurements were taken from distinct samples or whether the same sample was measured repeatedly
☐	☒	The statistical test(s) used AND whether they are one- or two-sided<br>*Only common tests should be described solely by name; describe more complex techniques in the Methods section.*
☒	☐	A description of all covariates tested
☐	☒	A description of any assumptions or corrections, such as tests of normality and adjustment for multiple comparisons
☐	☒	A full description of the statistical parameters including central tendency (e.g. means) or other basic estimates (e.g. regression coefficient) AND variation (e.g. standard deviation) or associated estimates of uncertainty (e.g. confidence intervals)
☐	☒	For null hypothesis testing, the test statistic (e.g. *F*, *t*, *r*) with confidence intervals, effect sizes, degrees of freedom and *P* value noted<br>*Give P values as exact values whenever suitable.*
☒	☐	For Bayesian analysis, information on the choice of priors and Markov chain Monte Carlo settings
☒	☐	For hierarchical and complex designs, identification of the appropriate level for tests and full reporting of outcomes
☒	☐	Estimates of effect sizes (e.g. Cohen's *d*, Pearson's *r*), indicating how they were calculated

Our web collection on statistics for biologists contains articles on many of the points above.

Software and code

Policy information about availability of computer code

Data collection	-Data were collected, in some instances, in Microsoft Excel version 2105<br>-Flow cytometry data were collected on a Bio-Rad S3E Cell Sorter using ProSort Software Version 1.6.0.12<br>-Observational data were collected in Benchling Electronic Notebook
Data analysis	- Prism v8 for Windows was used for analysis of data in some instances (AUC calculations, T-tests, and Figure generation)<br>- Microsoft Excel version 2105 was used for analysis of data in some instances (Figure and Table generation)<br>- Flow cytometry data was analyzed using FlowJo Single Cell Analysis Software v10.<br>- Boxplots were generated in RStudio version 1.2.5041 with R version 3.6.3 with the plotly R package (version 4.9.4.1).<br>- BioPython version 1.72 was used to generate Fig. 3c.<br>- Protein structures for Fig. 3 were generated using PyMOL version 2.4.0.<br>- Co-evolution analysis was performed using GREMLIN v2.1.<br>- Position specific scoring matrix (PSSM) was generated with PSI-BLAST using NCBI-BLAST 2.7.1.<br>- Rosetta package version 3.9 was used to generate the StlA homology model.

For manuscripts utilizing custom algorithms or software that are central to the research but not yet described in published literature, software must be made available to editors and reviewers. We strongly encourage code deposition in a community repository (e.g. GitHub). See the Nature Portfolio guidelines for submitting code & software for further information.

Data

Policy information about availability of data

All manuscripts must include a data availability statement. This statement should provide the following information, where applicable:

- Accession codes, unique identifiers, or web links for publicly available datasets
- A description of any restrictions on data availability
- For clinical datasets or third party data, please ensure that the statement adheres to our policy

All data generated or analyzed during this study are included in this published article (and the accompanying supplementary information files). Flow cytometry data (Fig. 1b) are provided as .fcs files in the compressed folder "Fig1b_fcs_files" in the Supplement. All other data are provided in the Supplemental file "Source data.xlsx." E. coli Nissle strain 1917 was obtained from DSMZ and used for the construction of engineered strains leading to the generation of SYNB1618 and SYNB1934. All strains described in this manuscript were derived from the same parental background. Engineered strains described in this manuscript can be made available subject to an MTA, which can be requested by contacting the corresponding authors. The complete genome sequence of SYNB1618 is available under BioProject ID: PRJNA482064. The complete genome sequence of SYNB1934 is available under BioProject ID: PRJNA749270

Field-specific reporting

Please select the one below that is the best fit for your research. If you are not sure, read the appropriate sections before making your selection.

[x] Life sciences [ ] Behavioural & social sciences [ ] Ecological, evolutionary & environmental sciences

For a reference copy of the document with all sections, see nature.com/documents/nr-reporting-summary-flat.pdf

Life sciences study design

All studies must disclose on these points even when the disclosure is negative.

Sample size	No in-life sample sizes were pre-determined. Sample size for the non-human primate study (n = 18) was based on the cost and availability of animals. The results derive from 12 NHP subjects subjected to 3 identical SYNB single dose experiments with a full week wash out in between the end of each experimental day. In each of the 3 experimental days, 6 NHP subjects were dosed with SYNB1618 or SYNB1934 and the data presented in Figure 5 is the combined results of the 3 experiments. Results of this study did yield highly significant results as reflected in the low p values obtained.
Data exclusions	Though 18 NHP subjects were utilized in the in-life study, only 17 data points were included in the urinary analysis of d5-hippurate excretion in the SYNB1934-treated study arm because one NHP subject failed to produce a urine sample over the 6 hour course of experimentation.
Replication	In vitro and in vivo activity of SYNB1618 and SYNB1934 is highly reproducible. Each of the key findings in Figure 5 have been repeated on multiple occasions and have yielded essentially identical results.
Randomization	For in-life studies involving non-human primates, subjects were weighted and randomized by weight into treatment groups.
Blinding	Blinding was not relevant to this study. However, analysis of in-life data was not performed by the same individuals that performed in-life portion of studies or the bioanalytical detection of the samples generated. Work with non-human primates was performed externally by a CRO. Analysis of NHP data was not performed by the same individuals that performed bioanalytical detection analysis.

Reporting for specific materials, systems and methods

We require information from authors about some types of materials, experimental systems and methods used in many studies. Here, indicate whether each material, system or method listed is relevant to your study. If you are not sure if a list item applies to your research, read the appropriate section before selecting a response.

Materials & experimental systems

n/a	Involved in the study	
[x]	[ ]	Antibodies
[x]	[ ]	Eukaryotic cell lines
[x]	[ ]	Palaeontology and archaeology
[ ]	[x]	Animals and other organisms
[x]	[ ]	Human research participants
[x]	[ ]	Clinical data
[x]	[ ]	Dual use research of concern

Methods

n/a	Involved in the study	
[x]	[ ]	ChIP-seq
[ ]	[x]	Flow cytometry
[x]	[ ]	MRI-based neuroimaging

Animals and other organisms

Policy information about studies involving animals; ARRIVE guidelines recommended for reporting animal research

Laboratory animals	Twelve male cynomolgus monkeys aged 2 to 5 years were used for non-human primate (NHP) studies. Animals ranged in weight from 2.5 - 4.0 kg). NHP studies were performed at Charles River Labs (Shrewsbury, MA)<br><br>Disposition of animals: Animals were in good health condition at the beginning of the studies.
Wild animals	No wild animals were used in this study
Field-collected samples	No field-collected samples were used in the study
Ethics oversight	Charles River Laboratory's Institutional Animal Care and Use Committee

Note that full information on the approval of the study protocol must also be provided in the manuscript.

Flow Cytometry

Plots

Confirm that:

- [x] The axis labels state the marker and fluorochrome used (e.g. CD4-FITC).
- [x] The axis scales are clearly visible. Include numbers along axes only for bottom left plot of group (a 'group' is an analysis of identical markers).
- [x] All plots are contour plots with outliers or pseudocolor plots.
- [x] A numerical value for number of cells or percentage (with statistics) is provided.

Methodology

Sample preparation	All flow cytometry data was performed on pools of engineered E. coli cells expressing GFP, with sorting of sub-populations based on level of GFP fluorescence
Instrument	Bio-Rad S3E Cell Sorter
Software	Prosort Software Version 1.6.0.12
Cell population abundance	This was a directed evolution experiment that was coupled to a fluorescent readout. Within a bacterial culture containing a mixed pool of mutants, the population of bacteria expressing the highest level of GFP fluorescence was predicted to be the desirable fraction of cells for further analysis. Successive top 1% sorting (top 1% of the cells exhibiting the highest level of GFP signal fluorescence) were isolated and used for further phenotypic analysis. No conclusions about specific outcome of the directed evolution were based solely off of the flow cytometry used in these studies, merely, it was a tool to sort populations for deeper analysis
Gating strategy	See above. Flow cytometry and bacterial cell sorting was used in this study to isolate populations of engineered bacteria for phenotypic analysis. The hypothesis for the strategy employed in these experiments was that the cells expressing the highest level of GFP fluorescence would constitute the fraction of cells containing proteins of interest. Therefore, the strategy was to selectively sort the top 1% fraction of cells expressing the highest level of GFP. This population was sorted again to isolate the top 1% of GFP-expressing strains from that population ("top 1% - top 1%").Prior to selection based on GFP (FITC-A), cells were gated based on cell size (SSC-A vs. FSC-A) and for doublet exclusion (FSC-H vs. FSC-A). A schematic of the gating strategy is shown in Fig. 2, and an example from the ProSort software can be found in Supplementary Fig. 6.

- [x] Tick this box to confirm that a figure exemplifying the gating strategy is provided in the Supplementary Information.
