# Supplementary material for: Improvement of a synthetic live bacterial therapeutic for phenylketonuria with biosensor-enabled enzyme engineering
Source: Nat Commun. 2021 Oct 28;12:6215. doi: 10.1038/s41467-021-26524-0 (PMC8553829; doi:10.1038/s41467-021-26524-0)
Supplement: Supplementary file 5 — Description of Additional Supplementary Files [file 41467_2021_26524_MOESM5_ESM.pdf]

Title: Supplementary Data 1

Description: A table containing all primers used for construction of PAL library
